# HIV self-testing in the real world is acceptable for many: post-test participant feedback from the GetaKit study in Ottawa, Canada

**DOI:** 10.1177/17449871221137761

**Published:** 2022-12-14

**Authors:** Patrick O’Byrne, Alexandra Musten, Nikki Ho

**Affiliations:** Nurse Practitioner and Fellow of the American Academy of Nursing, School of Nursing, University of Ottawa, Canada; School of Nursing, University of Ottawa, Canada; Registered Nurse, School of Nursing, University of Ottawa, Canada

**Keywords:** HIV, self-testing, real-world study, user feedback

## Abstract

**Background:**

HIV self-testing is the latest strategy to improve access to testing, diagnosis and treatment. Such strategies are beneficial due to the improved individual- and population-level health outcomes that emerge from early HIV diagnosis.

**Aims:**

While most research shows that HIV self-testing is acceptable and feasible, yielding higher numbers of first-time testers and positivity rates, compared to clinic-based testing, little evidence exists outside low- and middle-income countries about such testing.

**Methods:**

We implemented GetaKit.ca, a website through which eligible participants could register for and obtain an INSTI® HIV self-testing to their home, and then report the result back.

**Results:**

Those who returned to the website were asked to complete a post-test survey, which had a low response rate (42%), but identified satisfaction scores of 92%. Notably, 5% of testers sought in-person care after ordering the self-test, and only 80% of participants agreed that the INSTI® HIV self-test was easy to use.

**Conclusions:**

Participants provided tangible solutions to improve this test, which we feel are easy to incorporate and essential to maintain HIV self-testing efforts.

## Introduction

Most research has found that not only do participants prefer HIV self-testing over clinic-based testing, but also that they report higher satisfaction scores with self-testing (Bjørnshagen et al., 2020; Johnson et al., 2017; Laprise & Bolster-Foucault, 2021; McGuire et al., 2021; Rutstein et al., 2017; Witzel et al., 2020). These studies have also found that HIV self-testing yielded higher positivity and uptake rates among first-time testers, compared to traditional clinical testing (Bjørnshagen et al., 2020; Johnson et al., 2017; Laprise & Bolster-Foucault, 2021; McGuire et al., 2021; Rutstein et al., 2017; Witzel et al., 2020). This elevated preference for, satisfaction with, and uptake of, HIV self-testing signals that this testing modality may be an ideal method to decrease the number of persons who are unaware they are HIV positive (Witzel et al., 2020).

Such findings about expanding testing to first-time testers and higher positivity rates are important because early HIV diagnosis can lead to treatment and subsequent improvements in health status for HIV-positive persons and decreases in onward HIV transmission to HIV-negative persons (Novelli et al., 2020; Rutstein et al., 2017). In other words, diagnosis and access to care have both individual- and population-level benefits (Rutstein et al., 2017).

The extant literature, however, is not without shortcomings, as most studies occurred either in controlled clinical settings (Bwana et al., 2018; Galli et al., 2021; Majam et al., 2021) – with participants trained and/or observed doing self-tests – or in low- or middle-income countries. Many of these studies, moreover, used oral swabs, rather than finger-prick blood sample tests – which is an important distinction, as there is an identified preference for oral swabs (Stevens et al., 2018). As such, a major knowledge gap currently exists about end-user feedback regarding HIV self-testing using finger-prick methodologies in the real-world higher-income countries.

To add to current understandings about HIV self-testing, we undertook the first mail-out HIV self-testing pilot in Canada using the INSTI® HIV self-test (bioLytical Industries, 2021), which was a finger-prick blood sample test. To implement this study, we established GetaKit.ca, an online platform where persons could register, complete an HIV self-assessment and order a self-test to their home or other designated address. Our goal for this study was to observe the outcomes associated with this pilot and to obtain user feedback. While we have previously published on GetaKit implementation, preliminary data and invalid results (O’Byrne et al., 2021a, 2021b, 2022), this paper is the first report on participants’ experiences of, and feedback about, participating in HIV self-testing through the GetaKit study.

## Methods

GetaKit is a prospective open cohort observational study, which operated in multiple phases. Phase 1 was the feasibility pilot in Ottawa. Phase 2 involved the scale-up of GetaKit to the province of Ontario. Phase 3 involved the addition of COVID self-tests in Ontario. Phase 4 involved the addition of full sexually transmitted infection (STI) testing in Ottawa. This paper describes phase 1 of this study.

For phase 1, to be eligible, participants had to be >18 years old, living in Ottawa and not taking HIV pre-exposure prophylaxis; participants could neither have a diagnosed bleeding disorder nor be enrolled in an HIV vaccine trial. Those who were eligible in the study catchment area could complete a registration form, an HIV risk self-assessment and order a free HIV self-test, unless deemed not at-risk based on the self-assessment. The system operated through the website (GetaKit.ca) and required two-factor authentication using a cell phone for access. Delivery of HIV self-tests through the GetaKit study took 24–48 business hours.

We used the bioLytical INSTI® HIV self-test^10^, as it was the only HIV self-test licensed by Health Canada. This test was a ‘single use, rapid, flow through in vitro qualitative immunoassay’ to detect HIV. This test involved multiple steps: (1) collecting 50 mcg of fingerstick blood (estimated as one whole drop of blood), (2) mixing the drop of blood in diluent 1, (3) pouring diluent 1 into the test membrane and allowing it to flow through the membrane test disc, (4) pouring diluent 2 into the test membrane and allowing it to flow through, (5) pouring diluent 3 into the test membrane and allowing it to flow through and (6) interpreting the qualitative results that appear. Results appear as blue dots on a white background within 1 minute of testing and must be read within 1 hour of testing. A single control dot signals a negative result; two blue dots signal a positive result; and no blue dots or only a test dot signals an invalid result.

Participants were not required to report their HIV self-test results back to GetaKit, but were invited to do so, with email reminders sent at days 7 and 14 after they received their self-test by mail. Participants who returned to the website to report their result were asked to complete a non-obligatory post-test survey, for which they received a gift card. The post-test survey included questions about reasons for obtained HIV self-testing and experiences with the test ordering, test completion and results interpretation. One additional question sought open-ended feedback on experiences and feedback. See Table 1 for a list of questions.

**Table 1.** Post-test questions.

**Q1. Why did you use HIV self-test(s)? Check all that apply.** 1. It was more convenient than getting tested by a doctor or at an HIV testing site 2. It was more private than getting tested by a doctor or at an HIV testing site 3. I didn’t want other people to know I am testing 4. To test together with someone, before having sex 5. To test myself, before having sex 6. A sexual partner asked me to take a HIV self-test 7. Other reason. Please specify: ______________________ 8. prefer not to answer					
**Q2. Where did you get tested using the study HIV self-test kit? Choose all that apply.** 1. Home 2. A friend’s place 3. Other location. Please specify______________					
**Q3. Tell us about your experience with HIV self-test test received from this study**					
	Strongly Agree	Agree	Neutral	Disagree	Strong Disagree
It was easy to order the kit online					
The instructions were easy to follow					
Collecting the blood sample was easy					
Interpreting the results of the kit was easy					
Overall, I was satisfied with the process of ordering and using the kit					
I would recommend the kit to a friend					
**Q4. What could improve the experience of ordering and using the HIV self-test kit?**					

Data collection occurred via GetaKit.ca, through which all eligibility and registration data were stored. Data could be downloaded into an MS Excel file. For this paper, we reviewed the data in two ways.

First, the numerical data were analysed for frequencies and percentages. Specifically, we tallied the number of persons who ordered a self-test, reported their results back on our website and completed a post-test survey. We used the denominator of eligible participants to determine the percentages for these variables. For the post-test survey, we calculated frequencies and percentages for the first three questions in the same fashion.

First, for the open-ended feedback in question four of the post-test survey, we employed Guest et al.’s (2010) pragmatic thematic analysis by identifying the main points within the feedback, clustering similar points and naming the commonality (theme) within the points we had clustered. To explain further, we took the participants’ open-ended feedback and reviewed the text multiple times to gain familiarity and to identify the main point(s) within the data. We then listed these ‘main point(s)’ in a codebook. Next, we took these main point(s) (i.e. codes) and clustered those that were similar to create distinct topic areas in the data. We then reviewed these clusters to identify the commonality that linked the participants’ responses. We named the commonality we identified, thus creating themes, and reported on these themes as main findings.

The Research Ethics Board at the University of Ottawa approved this project, and the Ontario HIV Treatment Network funded the study.

## Results

From 20 July 2020 to 31 March 2021, 399 persons ordered an HIV self-test and completed a baseline survey through GetaKit.ca, of whom 57% (*n* = 228/399) reported test results back through the website; 73% (*n* = 167/288) of those who reported a result completed the post-test survey. The response rate for the post-test survey was 42% (*n* = 167/399) for everyone who ordered a test. Of reported results, 1 was positive, 177 were negative, 3 were ‘prefer not to report’ and 47 were invalid (for an invalid rate of 20% for reported results and 11% for all ordered tests).

Participant responses about HIV self-testing were favourable. Regarding reasons for using HIV self-testing, 77% (*n* = 128) stated convenience, 49% (*n* = 82) reported privacy and 4% (*n* = 6) said it was because of COVID restrictions. For location, 94% (*n* = 157) reported that they did the test at home. For ease of ordering, 93% (*n* = 156) agreed that the instructions were easy. For doing the finger-prick, 80% (*n* = 134) agreed that it was easy, 12% (*n* = 20) were neutral and 3% (*n* = 5) did not find it easy to complete. For reading the result, 87% (*n* = 146) found the test easy to interpret and 5% (*n* = 9) found it difficult to interpret. For overall satisfaction, 92% (*n* = 153) reported that they were satisfied. Interestingly, 5% (*n* = 8) stated that they sought out HIV testing at a clinic before doing the self-test.

For the open-ended feedback, 97 persons wrote answers. Thematic analysis identified six main domains. The first related to instructions, with participants requesting online tutorials. One specific item that was mentioned was the need to emphasise that the quantity of blood required exceeded expectations. The second was about delivery, requesting discrete packaging and a courier that would leave the kit in a safe drop location. The third was about availability, specifically focusing on expanding geographical distribution and increased targeted publicity to the groups most affected by HIV. The fourth related to supports for doing the test, results management and results interpretation. This theme had a particular emphasis on reading the results, especially with the dots being very faint. The fifth related to additional services, with requests for mail-out COVID self-tests and at-home STI testing. The sixth was about recommendations for technical changes to the kit, which included adding a second lancet, an alcohol swab and making it easier to get the drop of blood into the small opening of the test vial. Other feedback focused on the complexity of the test, with the large number of items included. See Table 2 for a summary of example quotes for each identified theme.

**Table 2.** Examples of feedback.

Theme	Example feedback
Instructions	•The instructional pamphlet is a bit overwhelming in terms of its content •Video tutorial online, especially for blood drawing •Write down that the drop of blood needs to be considerably large. My first attempt was unsuccessful therefore I needed to order a second kit •I was unsure how much blood was needed, and it was hard to locate the dots. It is important to say good light is needed, and perhaps information on the colour of the dot would be helpful
Delivery	•More discreet packaging on the outside might be nice •Avoid using a courier that requires you to pick up the item off-site if you miss the initial delivery
Availability	•More publicity on how to get the test kit •Publicize it more! •Make it available for people in Québec •This element of the process is quite straight forward and easy. . . if you can access the Internet, know how to set up additional accounts and follow through. . . however, that is a high barrier to engagement
Supports	•Reading the results was a bit tricky. The test only showed one blue dot but there were also two white dots below, very faded. I found out reading other brochures that only blue dots are results – there was n’t any indication about how to interpret white dots but I assumed only the blue dots are relevant •I had a hard time reading the dot when it appeared •It's always nerve-wracking taking a test. I think the suggestions of how to deal with testing anxiety (for those like me) could be better highlighted •Show more examples how to interpret results. Also real photos could be helpful as opposed to pictograms
Additional services	•This is the future of testing. Now do one for COVID! :) •You have been very responsive. . . I cannot think of anything to improve (unless there are ways of home self-swabbing for other STBBI’s). •I’ve known my GP more than a decade.. I still opt for the public health clinic for my HIV/STI screening so I don't burden her office hours. . . it would be awesome if they were able to issue me a RX for an at-home-STI screening. I think this format of health screening is awesome
Technical changes	•Larger opening on bottle to drip blood in to. Hard to aim drops of blood when hands are shaking from anxiety •Increasing the diameter of opening for the bottle you drip your blood into might be helpful, was a little tricky •Using the lancet may be difficult for people who haven’t used one previously; it required a bit more force in comparison to the reusable lancets used for glucose monitoring. Otherwise, the kit was really straight forward •Make sure there’s clear indication that the C on the kit is on top. I was reading my results and thought they were invalid when they were negative •Perhaps a way to not spill the liquid. Bottle 2 should be designed differently •Include an alcohol swab for the Lansing •Only step I found a minor challenge was to get my drop of blood to actually fall off my finger into the small bottle

## Discussion

The post-test survey results from phase 1 of the GetaKit study identified that about half of participants returned to the website to report a result (57%) and complete a post-test survey (42%). The responses to this survey highlighted that, while most participants gave favourable feedback about the project, many made recommendations for improvement, namely suggesting additional support and instructions regarding how to perform the test and interpret results. As well, although most participants agreed that the test was easy to perform, many indicated that the instructions were inadequate and that the testing device would benefit from modifications, including the addition of an alcohol swab, fewer items in the kit and an easier process for entering blood into the device. Notably, a few (5%) participants sought HIV testing in a clinic before doing the HIV self-test. These results raise a few points for discussion.

First, aligning with the literature (Bwana et al., 2018; Galli et al., 2021; Majam et al., 2021), we identified a high level of reported satisfaction (over 90%) among participants who completed the post-test survey. Due to the low response rate, however, these results cannot be generalised. Moreover, we are unable to discern if participants’ favourable perceptions related to the option to do self-testing – which was novel in our jurisdiction as part of the GetaKit study – or about the INSTI® kit itself. It is equally unclear if our findings were affected by the COVID-19 pandemic, which mostly shuttered access to HIV testing in the research area during the study period. Participants may have therefore reported more favourably about GetaKit and HIV self-testing than they otherwise would have because this study enabled them to access testing when they otherwise could not do so, rather than because they found the self-test device or our project well designed and implemented. That 5% of participants reported finding the test result difficult to interpret, however, suggests that some dissatisfaction related to the device itself. In any case, these results highlight that mail-out HIV self-testing via online ordering is accepted by some.

Second, that another 5% of persons who completed the post-test survey obtained in-person testing before doing the self-test and that 20% of participants were neutral or did not agree that the test was easy to use raises concerns about the perception of HIV self-testing more broadly. While those who sought in-person testing duplicated service delivery, those who did not find the testing experience user friendly highlighted the existence of a non-favourable perception about HIV self-testing using the INSTI® device. While previous research suggests that HIV self-testing is a viable option to decrease the proportion of persons who are unaware they are HIV positive (Bjørnshagen et al., 2020; Johnson et al., 2017; Laprise & Bolster-Foucault, 2021; McGuire et al., 2021; Rutstein et al., 2017; Witzel et al., 2020), our findings suggest that further work is required to improve the experiences and perceptions of such testing. This would include incorporating participant feedback about how to improve the INSTI® test itself; for example, fewer components, easier to use vials and a simpler method to obtain the required 50 mcg drop of blood.

Third, that only 80% of participants found the test was easy to use and that only 87% found the result was easy to interpret might have contributed to the overall 11% invalid rate we observed. In the only study of the INSTI® HIV self-test in a high-income North American country, which occurred in a controlled clinical setting where participants were observed doing the self-test, Galli et al. (2021) identified an invalid rate of 5.6%, plus an additional 2.7% who could not interpret the results, yielding a test failure rate of 8.3%. In our real-world study, 1 in 10 to 1 in 20 participants did not agree that the testing or results interpretation processes were easy, supporting our assertion that while HIV self-testing can be done alone, it requires additional resources and supports to make it useful. In the absence of such supports, multiple tests will be required for some participants, testing opportunities will be missed for others, and the public perception and trust of such testing could be lost. We recommend that these issues are addressed promptly before the benefits of self-testing are undermined by test devices inadequacies.

Fourth, these results have implications for nursing practice. For one, they highlight that nurses should promote HIV self-testing as a viable sexual health strategy. Although our sample was non-representative, it did show that a subset of participants favourably viewed the HIV self-testing and the GetaKit ordering process. Tailoring these services to the groups who are most affected by HIV could help ensure access to testing without requiring increased in-person health service delivery. That is, nurses could use local HIV epidemiology to increase awareness about, and uptake of, HIV self-testing, which could increase testing among the members of the populations most affected by HIV without requiring additional in-person clinical services. Another important point in our results is that participants wanted more support and assistance. Sexual health and public health nurses could provide such services – acting as resources for persons who use HIV self-testing. The key point from this finding is that a subset of our participants did not want to test alone while self-testing. Instead, they wanted support and guidance while performing the test. Based on pre-existing knowledge, public trust and expertise in sexual health, nurses could perform this role. Lastly, our results suggest that nurses could help develop online platforms to deliver HIV testing to rural and remote communities. Delivery could occur through mail services or local depots for curbside pick-up. Online, telephone-based and local support services could also be developed to maximise the user experience. In short, these results highlight that nurses are well situated (1) to implement HIV self-testing projects that provide testing to persons with undiagnosed infections, and (2) to provide the support and counselling that some of our participants reported as lacking in our study of pure self-testing in real-world at-home settings.

### Limitations

These findings must be interpreted considering certain limitations. This project occurred during the COVID-19 pandemic when access to HIV testing was otherwise difficult to obtain. This may have increased uptake and favourable perception among those who responded. This study also had a very low response rate for the post-test survey, and while our results show the perceptions of the minority who completed this survey, they should likely not be extrapolated further. Lastly, our study used only one finger-prick HIV self-test, and reported findings, meaning our findings may be the artefact of self-report about the INSTI® HIV self-test specifically.

## Conclusion

Persons with access to the Internet and a cell phone, who have fixed addresses and a location to perform the HIV self-testing could benefit from online access – and a subset of these persons reported favourably about obtaining HIV self-testing through GetaKit.ca. While this finding is important, it must be interpreted considering that half of the study participants did not return to the GetaKit website to report results – possibly due to a reluctance to report or difficulties navigating the system – and that many others would not have been able to access this testing due to lower tech literacy, socioeconomic status (i.e., no or limited access to a cell phone or the Internet), or language barriers. As such, while maintaining online access to HIV self-testing is important, effort needs to be made to ensure access to testing does not exacerbate inequities by making testing most available to those with the most resources. HIV self-testing is a valuable tool to continue to access care and may possibly yield a diversion approach for those who can use these services, thus allowing for a re-orientation of in-person care toward persons who have the greatest access barriers otherwise. Through such various access points, we may be able to further decrease the number of persons with undiagnosed HIV.

**Key points for policy, practice and/or research**

- HIV self-testing is a new strategy to increase access and diagnosis.
- Most participants reported favourable views about HIV self-testing and our online registration and distribution system.
- Participants found the INSTI® HIV self-test to be complicated and requested additional information and supporting materials.
